# Supplementary material for: First Detection of NADC34-like PRRSV as a Main Epidemic Strain on a Large Farm in China
Source: Pathogens. 2021 Dec 29;11(1):32. doi: 10.3390/pathogens11010032 (PMC8778757; doi:10.3390/pathogens11010032)
Supplement: Supplementary file 1 [file pathogens-11-00032-s001.zip › Supplementary Material S1.pdf]

Supplementary material S1.

The number of deaths in each time period and the summary of testing for five pathogens

	total number								
	**date[a]**	**death**	**age range[b]**	**sample[c]**	**PRRSV[d]**	**PCV2[e]**	**AFSV[f]**	**CFSV[g]**	**PRV[h]**
**stage1**	0-15d	46	42-64d	46	8	41	0	0	0
**stage2**	16-30d	133	58-79d	69	14	50	0	0	0
	31-45d	154	73-94d	91	39	81	0	0	0
**stage3**	46-60d	42	88-109d	40	14	32	0	0	0
	61-75d	20	103-124d	20	3	15	0	0	0
	76-90d	15	118-139d	15	5	13	0	0	0
	91-105d	1	133-154d	1	0	1	0	0	0
	106-120d	1	148-169d	1	0	1	0	0	0
	121-135d	0	163-184d	0	0	0	0	0	0
	136-150d	0	178-199d	0	0	0	0	0	0
**Total**	0-150d	412	42-199d	283	83	234	0	0	0

[a]date: time after piglets enter the fattening farm, day(d)

[b]age range： age range of dead pigs

[c]sample: number of samples collected

[d]PRRSV: porcine reproductive and respiratory syndrome virus

[e]PCV2: porcine circovirus 2

[f]AFSV: african swine fever virus

[g]CFSV: classical swine fever virus

[h]PRV: pseudorabies virus
